# Staircase Quantum Dots Configuration in Nanowires for Optimized Thermoelectric Power

**DOI:** 10.1038/srep31974

**Published:** 2016-08-23

**Authors:** Lijie Li, Jian-Hua Jiang

**Affiliations:** 1 Multidisciplinary Nanotechnology Centre, College of Engineering, Swansea University, Bay Campus, Swansea, SA1 8QQ, UK; 2 College of Physics, Optoelectronics and Energy, & Collaborative Innovation Center of Suzhou Nano Science and Technology, Soochow University, 1 Shizi Street, Suzhou 215006, China

## Abstract

The performance of thermoelectric energy harvesters can be improved by nanostructures that exploit inelastic transport processes. One prototype is the three-terminal hopping thermoelectric device where electron hopping between quantum-dots are driven by hot phonons. Such three-terminal hopping thermoelectric devices have potential in achieving high efficiency or power via inelastic transport and without relying on heavy-elements or toxic compounds. We show in this work how output power of the device can be optimized via tuning the number and energy configuration of the quantum-dots embedded in parallel nanowires. We find that the staircase energy configuration with constant energy-step can improve the power factor over a serial connection of a single pair of quantum-dots. Moreover, for a fixed energy-step, there is an optimal length for the nanowire. Similarly for a fixed number of quantum-dots there is an optimal energy-step for the output power. Our results are important for future developments of high-performance nanostructured thermoelectric devices.

Thermoelectric energy harvesting has the reverse effect as opposed to the thermoelectric refrigerator^1^,^2^, and has been studied extensively in recent decades^3^,^4^,^5^,^6^. Right from the invention of the Seebeck and Peltier effects up to now, people have been using doped semiconductor materials with the aim of increasing the electrical conductivity and reducing thermal conductivity for a higher figure of merit. Figure 1a,b show conventional two-terminal Seebeck thermoelectric energy harvester in its normal and unfolded geometries, respectively. The configuration in Fig. 1b is very similar to *p*-*n* junction for solar cells. However, the metallic contacts in the middle remove the junction barrier and enables elastic thermoelectric transport. Although Fig. 1b reveals the similarity and difference between a thermoelectric engine and a solar cell, the mechanism that accounts for the significant difference of the two devices in efficiency (i.e., solar cells have much higher efficiency compared with thermoelectric engines) has not been uncovered. It was found only recently that a *p*-*n* junction thermoelectric engine based on hot-phonon-assisted interband transition can have considerably augmented thermoelectric efficiency and output power compared to conventional two-terminal thermoelectric devices with the same material. The intrinsic mechanism that distinguishes thermoelectric engines and solar cells in their efficiency and output power is then revealed as akin to inelastic transport processes in a three-terminal geometry^7^,^8^ (Fig. 1c).

Other prototypes of three-terminal inelastic thermoelectric devices include Coulomb coupled quantum-dots (QDs)^9^,^10^, phonon-assisted hopping in QD chains (or localized states in 1D or 2D systems)^11^,^12^,^13^,^14^,^15^, and inelastic thermoelectric transport across an electronic cavity promoted by mismatched resonant tunneling at the two-sides of the cavity^16^,^17^,^18^,^19^. In those devices thermal energy from the third, insulating terminal of phonon or electronic bath is converted to electrical energy between the source and the drain (and vice versa). It was found that to optimize the performance (efficiency and output power) the energy of the two QDs has to be above and below the chemical potential around 3*k*_*B*_*T*, respectively. Experimental developments on three-terminal inelastic thermoelectric devices in mesoscopic systems at low-temperature were established recently^20^,^21^,^22^, attracting more and more researches in the field^8^.

In this work we focus on phonon-assisted hopping thermoelectric transport in a three-terminal thermoelectric energy harvester. Our main concern is to optimize the output power of such a device by tuning the energy configuration of a chain of QDs embedded in a nanowire. Such a scheme can be used to form a macroscopic thermoelectric device, since many parallel nanowires can be assembled together and the nanoscale thermoelectric engines can be connected in series. We compare the power factor (density) *P* = *σS*^2^ for these configurations consisting of many serially connected nano- thermoelectric engines along the *x* direction (while in *y*-*z* directions there are many parallel nanowires, see Fig. 2). Specifically, we focus on two configurations: (1) in each nano-engine there is only a single pair of QDs; (2) in each nano-engine there are a chain of *N* QDs with staircase configuration of energy (see Fig. 1d). We emphasize that the density of QDs along *x* direction is the same for all these situations and the only difference here is the energy configuration. We show that the power factor is largest for staircase energy configuration (the main focus of this paper). Particularly we study the dependence of power factor on the number *N* and the energy-step *dE* for each hopping. We find that for a given *dE* there is an optimal number *N* that maximizes the power factor. Our findings reveal important information for future design of inelastic thermoelectric devices.

## Three-Terminal Thermoelectric Transport for Nanowire Quantum-Dots

**Figure 1:**
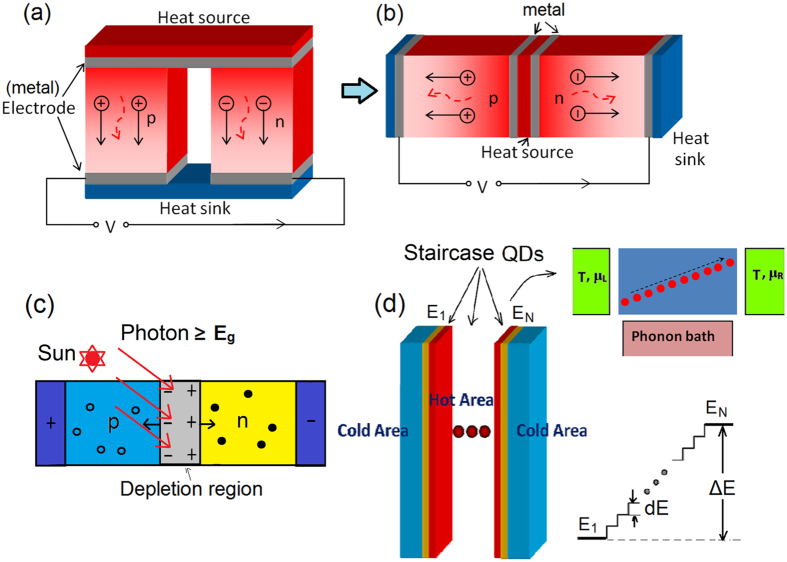
(**a**) Schematic of the conventional thermoelectric energy harvester that converts heat to electricity. (**b**) Unfolded geometry of the thermoelectric energy harvester. (**c**) Working principle of a solar cell, a three-terminal device akin to inelastic (i.e., photon absorbing) processes. (**d**) Schematic of staircase quantum-dots thermoelectric harvester. Heat from hot phonon bath is exploited to generate electricity via phonon-assisted electron hopping in a chain of quantum-dots with staircase energy configuration. The energy diagram for this device is illustrated as well. Each energy step is *dE*. For a chain of *N* quantum-dots the total energy difference is Δ*E* = (*N* − 1)*dE*.

The three-terminal thermoelectric energy harvesting device reported here is composed of two electrodes on the left and right sides under room-temperature environment, and a central region comprising QDs heated by the external phonon bath (Fig. 1d). By absorbing the phonon energy electrons hop from one QD to another which leads to electrical current against the voltage gradient. These are the processes that convert thermal energy from phonon bath to electrical energy^7^. For realistic devices operating at room-temperature and above, electron hopping is efficiently assisted by scattering with optical phonons. Optical phonon scattering transfer a considerable amount of energy, ranging from 10 meV to 120 meV for various materials^23^. In addition, electron-optical-phonon scattering time can be as short as 0.1 ps, leading to collision broadening as large as 10 meV^24^. These features make optical-phonon-assisted hopping thermoelectric transport as promising mechanism for powerful and efficient thermoelectric energy conversion.

**Figure 2:**
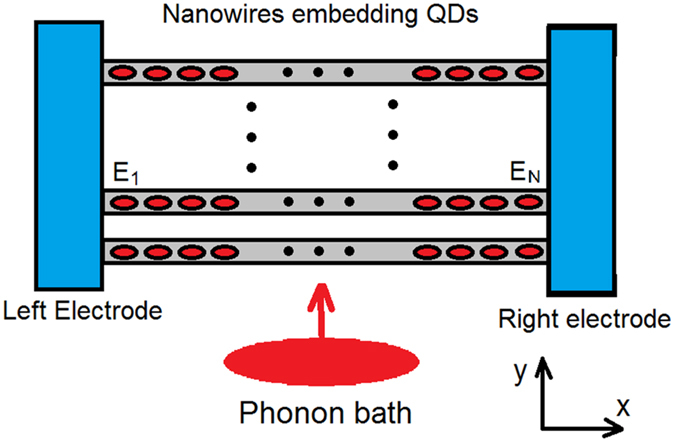
Schematic diagram of the thermoelectric energy harvester based on series of quantum dots embedded in parallel nanowires.

We shall consider many serially connected nano- thermoelectric engines along the *x* direction, while in *y*-*z* directions there are many parallel nanowires, as shown in Fig. 2. Specifically, each QD is of length *l*_*qd*_ = 6 nm along *x* direction. The distance between adjacent QDs is *d* = 6 nm. The probability of finding an electron outside the QD decays exponentially with the distance away from it with a characteristic length *ξ* = 2 nm. A simplified treatment based on Fermi golden rule yields the following hopping transition rate from QD *i* to QD *j*,


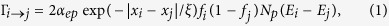


where the factor of two comes from spin-degeneracy, *α*_*ep*_ = 10 meV characterizes the strength of electron-phonon scattering, *f*_*i*_ and *f*_*j*_ are the probability of finding electron on QDs *i* and *j*, respectively. The *x* coordinates of the two QDs are *x*_*i*_ and *x*_*j*_, respectively, while their energies are *E*_*i*_ and *E*_*j*_, respectively. Here the phonon distribution function is given by


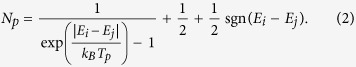


In our thermoelectric energy harvester, the phonon bath has temperature higher than the electrodes, i.e., *T*_*p*_ > *T*. The heat from the phonon bath is then converted into electricity. The electron distribution in each QD can be described by a Fermi distribution


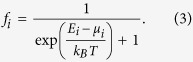


From the above, the electric current flowing from QD *i* to QD *j* is given by


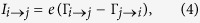


with *e* being the charge of a single electron. The linear conductance of electric conduction between QDs *i* and *j* is given by


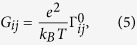



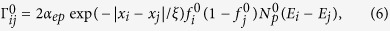


where 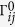 is the transition rate at equilibrium and the superscripts 0 denote equilibrium distributions. Hence each pair of QDs form a resistor with conductance *G*_*ij*_. Hopping conduction is mapped to conduction in network of resistance (i.e., the Miller-Abrahams network^25^). Such method is generalized to three-terminal hopping conduction in refs 11,12. Thermoelectric transport through the system is calculated by solving the Kirchhoff current equation, i.e., the total current flowing into QD *i* is equal to the total current flowing out of QD *j* for the Miller-Abrahams network^12^. In this fashion the electrochemical potentials at each QD. i.e., *μ*_*I*_’s, are determined numerically via the method presented in ref. 12.

We shall consider a chain of *N* QDs with staircase energy configuration. Each energy step is *dE* = *E*_*i*+1_ − *E*_*i*_ (we focus on the situation with *dE* > 0). The total energy difference is Δ*E* = (*N* − 1)*dE*. The first QD has energy *E*_1_ = −Δ*E*/2, while the last QD has energy *E*_*N*_ = Δ*E*/2. Here the energy we referred to is the energy of the lowest two degenerate electronic levels (i.e., spin-up and spin-down) of the QD. Higher levels in the QDs are ignored due to their much higher energies as we consider small QDs here. *N* = 2 is the case with a single pair of QDs in a nano-thermoelectric engine. The energy configuration is chosen to have particle-hole symmetry, which has been proven to be best for thermoelectric performance as shown in refs 16,18.

It is noted that differing from variable range hopping between randomly localized states in nanowires or higher dimensional systems, the staircase energy configuration always favours the nearest neighbour hopping. This is because hopping to farther neighbour QDs costs larger energy gap and longer distance simultaneously. In contrast, in variable range hopping, the nearby neighbours may have larger energy differences compared to farther localized states. Optimization of the hopping distance in 1D localized system leads to the Mott’s law^26^ in non-interacting electron systems (with slight modifications^27^).

It is necessary to mention that the Fermi golden rule requests that the energy difference *dE* between the two electronic states must be the same as the optical phonon energy (i.e., microscopic energy conservation). We are interested only in the range with *dE* ∈ (10, 120) meV, which can be realized in III-V, II-VI, VI semiconductors. Considering the electron-optical-phonon scattering rate for most of those semiconductors are around 0.1 ps. Our model calculation hence captures the main physics of the system. We mention that acoustic-phonon scattering near the Debye frequency is also very efficient. In our calculation, modifying *dE* may need to be fulfilled by changing the materials for nanowire-QDs. Nevertheless, our study reveals for a given *dE* (i.e., a given material) the number of QDs in a single nanowire that optimizes the power factor, as well as how such an optimal number varies with *dE*. These information are useful for future material design of nanowire-QD thermoelectric devices.

Beside the inelastic hopping conduction, there is also elastic transport through the system. The elastic transport defines pure electron quantum tunneling mechanism between QDs and electrodes. A resonant tunneling mechanism is exploited to describe such conduction process. Note that since we consider QDs with considerably large energy differences (much larger than coupling between quantum dots) sequential tunneling between QDs is suppressed. The dominant contribution comes from single QD resonant tunneling^12^, where each QD forms one of such resonant tunneling conduction channel independently. Hence the elastic conduction contributes to the electric current via


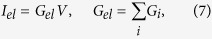



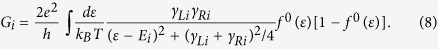


Here *V* is the voltage across the source and drain electrodes, *h* is the Planck constant, *G*_*el*_ denotes the elastic conductance, *f*^0^(*ε*) = 1/[exp(*ε*/(*k*_*B*_*T*)) + 1] (we set the electrochemical potential at equilibrium as energy zero). The tunnel coupling between the QD *i* and the left (right) electrode is *γ*_*Li*_ (*γ*_*Ri*_). We shall set the coordinate of the left electrode as *x* = 0, while the right electrode has *x* = *L*_*tot*_ with *L*_*tot*_ = *Nl*_*qd*_ + (*N* − 1)*d* + 2*l*_*b*_ where *l*_*b*_ is the distance between the first (last) QD and the left (right) electrode. The tunnel coupling is hence *γ*_*Li*_ = *t*_0_ exp(−*x*_*i*_/*ξ*) and *γ*_*Ri*_ = *t*_0_ exp(−(*L*_*tot*_ − *x*_*i*_ − *l*_*qd*_)/*ξ*) with *t*_0_ = 100 meV that characterizes hybridization energy of closely coupled QDs. We emphasize that the elastic current *I*_*elas*_ does not vary with the temperature of the phonon bath *T*_*p*_ since it originates purely from the quantum tunneling instead of coupling with phonons. In fact, in our thermoelectric engine, elastic conduction dissipates the electric energy generated by inelastic hopping into Joule heating.

Thermoelectric transport in our system the linear-response regime can be described by the coupled electric and heat conduction equation


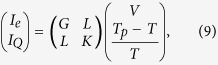


where *G* = *G*_*in*_ + *G*_*el*_ with *G*_*in*_ being the inelastic conductance. It was shown in ref. 12 that 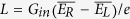 where 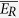 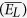 is the average energy of electrons entering into the right (left) electrode. For instance, hopping thermoelectric transport in a single pair of QDs gives *L* = *G*_*in*_ (*E*_2_ − *E*_1_)/*e*. Hopping for a chain of *N* QDs with staircase energy configuration yields *L* = *G*_*in*_ (*E*_*N*_ − *E*_1_)/*e*. We emphasize that elastic tunneling does *not* contribute to the Seebeck effect here, which is the essential difference between three-terminal and conventional thermoelectric effects. The Seebeck coefficient for the phonon-driven three-terminal thermoelectric effect is then


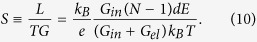


**Figure 3:**
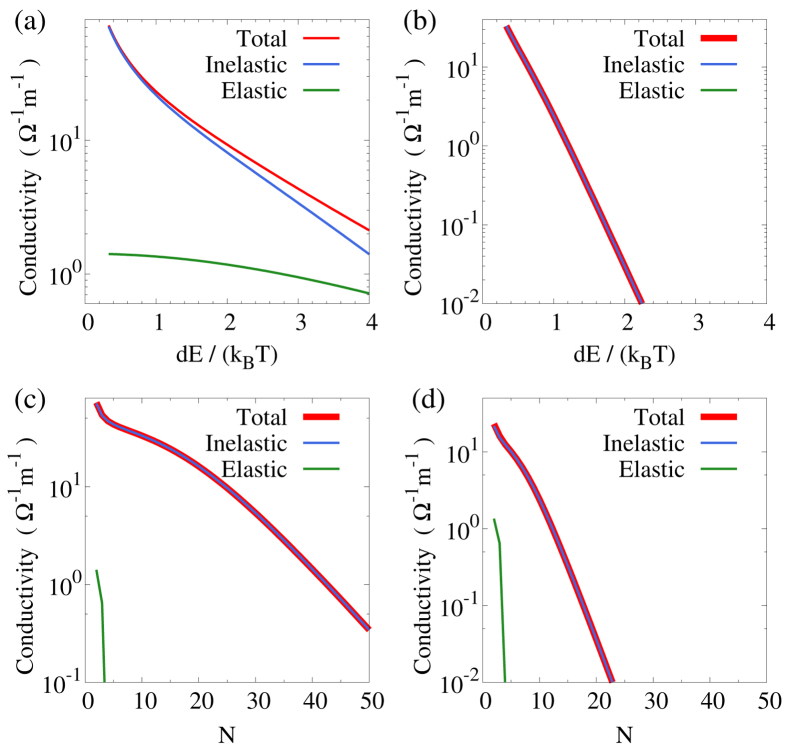
(**a**,**b**) Inelastic, elastic, and total conductivity as functions of *dE* for (**a**) a single pair of QDs and (**b**) a chain of *N* = 10 QDs with staircase energy configuration. The range of *dE* is between 10 and 120 meV. *k*_*B*_*T* = 30 meV. (**c**,**d**) Inelastic, elastic, and total conductivity as functions of the number of QDs in a single nano-device with staircase energy configuration for (**c**) *dE* = 10 meV and (**d**) *dE* = 30 meV.

For our system to work as an thermoelectric energy harvester, the inelastic conduction should dominate over the elastic conduction. The inelastic, elastic and total conductivity are plotted in Fig. 3(a) for a nano thermoelectric harvester with a single pair of QDs as functions of energy step *dE* for *dE* ∈ (10, 120) meV. Both the inelastic and elastic conductivity decreases with increasing *dE*. The elastic conductivity is reduced as the first QD has lower energy below the electrochemical potential while the second QD is higher above the electrochemical potential, leading to less effective conduction. The inelastic conductivity is also reduced due to the larger thermal activation energy *dE* and exponentially decreased phonon number 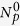. Therefore at very large energy difference *dE* the elastic conductivity may be more important. In reality, the elastic conduction also dominates in the small *dE* regime, which we ignored in this study. For a chain of QDs with *N* = 10, the results in Fig. 3(b) shows that the elastic conduction is much reduced, since tunneling over a longer distance is exponentially suppressed. The conductivity is then dominated by inelastic hopping in long chains of QDs.

Next we examine the conductivity as a function of the length of the chain. We show the results in Fig. 3(c,d) for *dE* = 10 meV and 30 meV, respectively. As the number of QDs increases both the inelastic hopping conductivity and elastic tunneling conductivity decreases. However, the elastic conductivity decreases much rapidly. The initial decay of hopping conductivity is sub-exponential, since increase the number of hopping is similar to increase the number of resistors. However, for large *N*, as the energy of the first (last) few QDs is much lower (higher) than the electrochemical potential, the hopping rates are suppressed by the exponentially small availability (occupation) of the final (initial) state. The decrease of conductivity at large *N* is hence exponential. Such exponential decrease become stronger for larger *dE* = 30 meV as shown in Fig. 3(d).

## Power Factor for Different Energy Configurations

We then study the power factor *P* = *σS*^2^ for various energy step *dE* and length of the QDs chain *N*. We remark again that for all situations the density of QDs is the *same*, according to our geometry of the nanowire QDs. The conductivity is calculated via *σ* = *Gl*/*A* where *l* and *A* are the length and area of a single nano thermoelectric engine. Here the area is determined by the density of nanowires as *A*^−1^ = 10^15^ m^−2^ 28,29. By focusing on the scale independent conductivity *σ* and power factor *σS*^2^ we are able to discuss ways of optimizing the power factor by engineering each nano thermoelectric element. In this way, the variation of the power factor *σS*^2^ is a sole consequence of the energy configuration (rather than geometry) in each nano thermoelectric engine.

**Figure 4:**
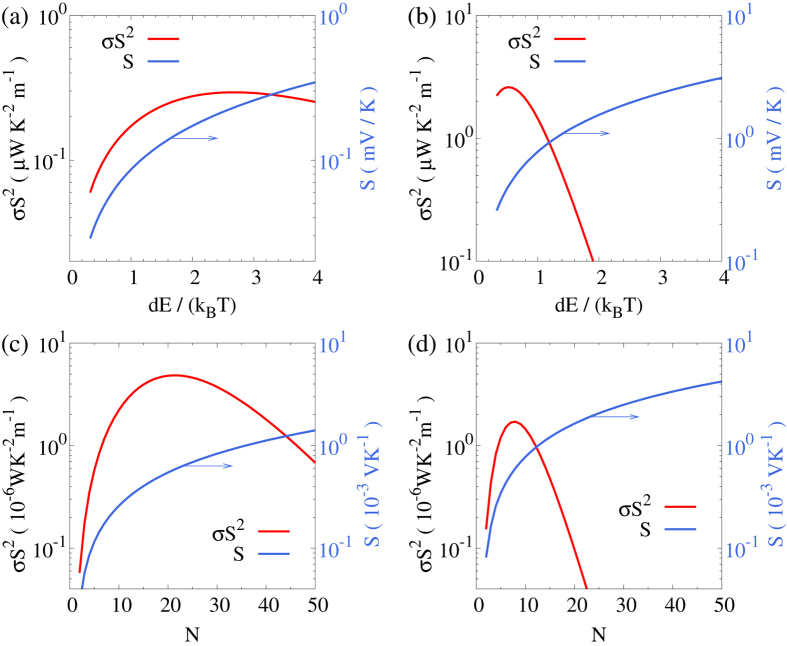
(**a**,**b**) Power factor *P* = *σS*^2^ and Seebeck coefficient *S* as functions of energy step *dE* for (**a**) a single pair of QDs and (**b**) a chain of *N* = 10 QDs with staircase energy configuration. (**c**,**d**): Power factor *P* = *σS*^2^ and Seebeck coefficient *S* as functions of the number of QDs in a single nano-device with staircase energy configuration for (**c**) *dE* = 10 meV and (**d**) *dE* = 30 meV. *k*_*B*_*T* = 30 meV.

In Fig. 4(a) we show the dependences of power factor *σS*^2^ and the Seebeck coefficient *S* on the energy step *dE* for a nano device with a single pair of QDs. It is found that the Seebeck coefficient *S* increases monotonically with the energy step *dE*, which is consistent with Eq. (10). As a consequence of competition between the conductivity and the Seebeck coefficient, the power factor is optimized around *dE* = 3*k*_*B*_*T*. For a chain of *N* = 10 QDs, the power factor is maximized at a much lower *dE*, as shown in Fig. 4(b). This is due to the more rapid decay of the conductivity as shown in Fig. 3(b).

Similarly, the dependence of the number of QDs *N* for a given energy difference *dE* also has a peak, as shown in Fig. 4(c,d). In Fig. 4(c) we plot the power factor *σS*^2^ and the conductivity *σ* as functions of the number of QDs *N* in a single nano device for *dE* = 10 meV. The power factor is maximized at *N* = 21. This maximum also appears as a consequence of the competition of the conductivity and the Seebeck coefficient when the number *N* is increased. The Seebeck coefficient increases as the total energy difference Δ*E* = (*N* − 1)*dE* increases, while the conductivity decays exponentially with the number of QDs for large *N*. Since such exponential decay of conductivity is more severe for larger energy step *dE*, the maximum appears at a smaller number of QDs *N* for *dE* = 30 meV, as shown in Fig. 4(d).

**Figure 5:**
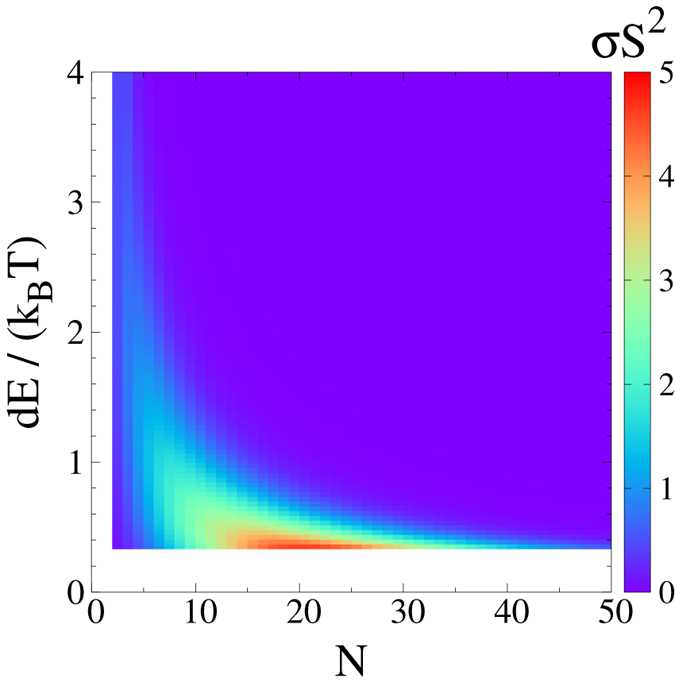
Power factor *P* = *σS*^2^ as a function of the energy step *dE* and the number of QDs in a single nano-device with staircase energy configuration. *k*_*B*_*T* = 30 meV. Other parameters are specified in the main text.

To have a global view of the dependence of the power factor on the energy configuration of QDs, we plot the *σS*^2^ for various *N* and *dE* in Fig. 5. It is seen that for each *dE* there is an optimized *N* at which the power factor is maximized. For smaller *dE* the optimal *N* is larger. More importantly, the maximal power factor is greater. Our study thus reveal the optimal energy configurations for powerful three-terminal thermoelectric energy harvester. In reality, it is important to find the energy *dE* that optimize the phonon-assisted hopping rate and the conductivity. Fixing such a *dE* one can find an optimal number of QDs *N* that form the maximal output power for a given material.

## Conclusion

We study the optimization of energy configurations of thermoelectric energy harvester assembled by many nano thermoelectric elements. Each nano device contains *N* QDs of staircase energy configuration with energy step *dE*. It is found that such energy configuration is *better* than the situation studied before: each nano thermoelectric element contains only *N* = 2 QDs. More importantly, we find that for each given energy step *dE* there is an optimal number of QDs *N* that maximizes the power factor. Such optimization yields higher output power when *dE* is smaller. Finally, we argue that our design is also better in thermoelectric power factor than hopping in a chain of QDs with random energy configuration. This is because the conductivity of such a random energy QDs chain is lower than that of the nano device with a single pair of QDs (when its parameters are optimized). On the other hand, the Seebeck coefficient is fluctuating around zero^12^, yielding relatively low Seebeck coefficient. Therefore, the power factor can be relatively lower as compared to the situation of assembled thermoelectric energy harvester with each nano-scale element contains a single pair of QDs. Our design of staircase energy configuration can have much better output power than the sigle-pair of QDs nano device. Therefore, our study is valuable for future design of nanostructured thermoelectric devices. Future study should also include the effect of parasitic heat conduction due to, e.g., phonon thermal conductivity, which may reduce the figure of merit, although it is usually much smaller in nanowires than in bulk materials.

## Methods

The power factor is calculated by computing the conductivity *σ* and Seebeck coefficient *S*. From Eq. (10), the essential quantities of interest are the conductivity for both inelastic and elastic transport processes. The conductivity for elastic processes are calculated via Eqs (7) and (8). The hopping conductivity is calculated via solving the Kirchhoff current equation for Miller-Abrahams resistor network numerically. The key quantities to be calculated are the “local voltage” *V*_*i*_ = *μ*_*i*_/*e* for all *i* labeling the QDs. According to ref. 12 the equations to be solved are


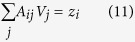


where


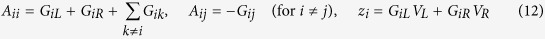


The left electrode has voltage *V*_*L*_ = *V*/2, while the right electrode has voltage *V*_*R*_ = −*V*/2. The conductance *G*_*iL*_ (*G*_*iR*_) is finite only when *i* labels the first (last) QD, and 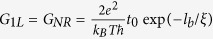. The hopping conductance between QDs are given by Eqs (5) and (6). By setting *V* = 0.01 and solving the above equation, we obtain *V*_*i*_ for all *i*. The electrical current flowing through the system due to hopping is then calculated via 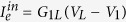 using the numerically obtained *V*_1_. The inelastic conductance of the nanowire-QDs system is then 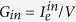.

## Additional Information

**How to cite this article**: Li, L. and Jiang, J.-H. Staircase Quantum Dots Configuration in Nanowires for Optimized Thermoelectric Power. *Sci. Rep.* **6**, 31974; doi: 10.1038/srep31974 (2016).
