# Corrigendum: Staircase Quantum Dots Configuration in Nanowires for Optimized Thermoelectric Power

**DOI:** 10.1038/srep33942

**Published:** 2016-09-22

**Authors:** Lijie Li, Jian-Hua Jiang

Scientific Reports 6: Article number: 3197410.1038/srep31974; published online: 08 23 2016; updated: 09 22 2016

In the original version of this Article, there were typographical errors in Affiliation 1 which was incorrectly given as ‘Multidisciplinary Nanotechnology Centre, College of Engineering, Swansea University, Bay Campus, Swansea, SA1 8QQ, UK.’ The correct affiliation is listed below:

Multidisciplinary Nanotechnology Centre, College of Engineering, Swansea University, Bay Campus, Swansea, SA1 8EN, UK.

This error has now been corrected in the PDF and HTML versions of this Article.

